# Technological interventions in European dementia care: a systematic review of acceptance and attitudes among people living with dementia, caregivers, and healthcare workers

**DOI:** 10.3389/fneur.2024.1474336

**Published:** 2024-10-02

**Authors:** Michele Sorrentino, Claudio Fiorilla, Michelangelo Mercogliano, Federica Esposito, Irene Stilo, Giuseppina Affinito, Marcello Moccia, Luigi Lavorgna, Elena Salvatore, Elisabetta Maida, Elisa Barbi, Maria Triassi, Raffaele Palladino

**Affiliations:** ^1^Department of Public Health, University “Federico II” of Naples, Naples, Italy; ^2^PhD National Programme in One Health Approaches to Infectious Diseases and Life Science Research, Department of Public Health, Experimental and Forensic Medicine, University of Pavia, Pavia, Italy; ^3^Department of Molecular Medicine and Medical Biotechnology, Federico II University of Naples, Naples, Italy; ^4^Multiple Sclerosis Unit, Policlinico Federico II University Hospital, Naples, Italy; ^5^Department of Advanced Medical and Surgical Sciences, University of Campania “Luigi Vanvitelli”, Naples, Italy; ^6^Meyer Children’s Research Institute, Meyer Children’s Hospital IRCCS, Florence, Italy; ^7^Interdepartmental Research Center in Healthcare Management and Innovation in Healthcare (CIRMIS), Naples, Italy; ^8^Department of Primary Care and Public Health, School of Public Health, Imperial College, London, United Kingdom

**Keywords:** Alzheimer, dementia, technology, acceptance, attitude, systematic review

## Abstract

**Background:**

Alzheimer’s and other neurodegenerative forms of dementia affect 8 million Europeans. Assistive technologies are suggested to reduce the burden of care and improve the quality of life of person living with dementia. Nonetheless, the acceptance and attitudes toward technological interventions pose challenges not only for people living with dementia and caregivers but also for healthcare workers. This review specifically aims to investigate how these key groups perceive and accept technology in European dementia care settings.

**Methods:**

This systematic review was conducted to identify studies, published between 2013 and 2023, that examined the acceptance and attitude of assistive technologies in Alzheimer’s and other dementia European settings, following the PRISMA guidelines. Rayyan AI was used for data extraction, and bias was assessed using the Mixed Methods Appraisal Tool.

**Results:**

Among the 1,202 identified articles, 13 met the inclusion criteria, revealing a prevailing positivity toward technological interventions in dementia care. Nonetheless, several barriers to adoption, including technological unfamiliarity, and specific dementia-related symptoms that complicate usage were identified. They also unveiled varying attitudes, influenced by factors such as familiarity with technologies, perceived usefulness, and the broader context of the COVID-19 pandemic which accelerated telemedicine and digital solution acceptance during restricted mobility and social distancing.

**Conclusion:**

Understanding attitudes toward technology in dementia care is crucial as it influences the adoption and utilization of tech-based interventions, impacting symptom management and quality of life. Addressing these attitudes through tailored interventions and education can enhance well-being and quality of life for people living with dementia, caregivers, and healthcare professionals.

## 1 Introduction

Alzheimer’s and other forms of neurodegenerative dementia constitute a complex set of progressive conditions that primarily affect older adults (1). Both are recognized as leading causes of disability in the elderly (2). In 2019, approximately 7,853,705 people were estimated to have Alzheimer’s or other dementia in Europe, with this figure anticipated to double by 2050 worldwide (3).

Nevertheless, a growing body of evidence suggests a declining trend during the last 25 years in dementia’s incidence, in Europe and United States (4). This drop may be attributed to increases in educational attainment and improvements in the management of cardiovascular disease and its associated risk factors (5). However, despite this positive trend, dementia has far-reaching impacts—physically, psychologically, socially, and economically—not only on persons living with dementia (PLWD) but also on their families and caregivers (6). Furthermore, dementia often results in a gradual decline in the ability to carry out daily tasks independently, contributing to social isolation and loneliness for both PLWD and their family caregivers (7).

Non-pharmacological interventions, particularly involving assistive technology, are increasingly recommended as primary treatments for dementia (8). Various technology-based interventions have been developed to manage dementia symptoms (9–11), reduce caregiver burden, and enhance patients’ quality of life (12–14). Nonetheless, older adults and their caregivers often have concerns regarding technology (15) and face considerable stress when introducing technological support (16).

These attitudes toward technology are influenced by personal characteristics, as well as technology-related factors and the social context (17). The most common barriers in the adoption of technology by older people are familiarity and access, need for assistance, trust, privacy implications, design, and physical issues with reduced dexterity and precision (18).

Furthermore, the COVID-19 pandemic exacerbated challenges for PLWD, disrupting routines, reducing cognitive stimulation, worsening neuropsychiatric symptoms and limiting social interactions (19–22). In fact, during the pandemic, access to support systems and non-pharmacological interventions for dementia management, crucial for combating loneliness and isolation (23, 24) was restricted during lockdowns (25). In this context, digital technologies have the potential to address these social connectivity issues by facilitating broader social connections (20, 26).

However, challenges remain in the use of technology by PLWD, emphasizing the need for tailored interventions and support mechanisms (27), as well as a pressing need to investigate how technologies can improve their daily lives (28). Furthermore, numerous other stakeholders play vital roles, including caregivers, healthcare organization managers, and technology suppliers (29, 30).

To the best of our knowledge, this systematic review is the first assessing acceptability and attitude toward technological intervention among European Alzheimer’s and dementia settings. This review is conducted in light of the growing recognition of the significance of assisting technology in improving the quality of life for patients, as well as caregivers and healthcare professionals, within dementia settings.

## 2 Methods

This systematic review analyzed studies focusing on acceptability and attitude toward technological intervention in European Alzheimer’s and dementia patients and was conducted following the Preferred Reporting Items for Systematic Reviews and Meta-Analyses (PRISMA) guidelines (31).

### 2.1 Study design

This systematic review analyzed studies focusing on acceptability and attitude toward technological intervention in European Alzheimer’s and dementia patients.

Including criteria were studies published in English, reporting original research (e.g., cohort studies, cross-sectional studies, case–control studies, or qualitative investigations), and conducted in Europe. We included studies focusing on Alzheimer’s and other forms of dementia, regardless of age, gender, race, and socioeconomic status, if formally diagnosed. Studies addressing other aspects of technology, such as efficacy, performance test, comparative test were excluded. Finally, studies were excluded if full text was not available.

This systematic review used the PICO framework to define the inclusion criteria, focusing on populations formally diagnosed with dementia or Alzheimer’s disease, along with both formal and informal caregivers and healthcare professionals. The interventions considered in this review involved the use of technological solutions, with the primary outcomes assessing attitudes and acceptance of these interventions. No specific comparison was made. The criteria to include or exclude an article are resumed in Table 1.

**Table 1:** Eligibility criteria.

Inclusion criteria	Exclusion criteria
**Population:** European setting People living with dementia or Alzheimer’s Disease with a formal diagnosis Informal caregivers of people living with dementia Formal caregivers of people living with dementia Healthcare professionals involved in dementia care Other stakeholders involved in dementia care **Intervention:** Assessment or search of: Attitudes toward Technology of one or more populations Acceptability of Technology or Technological Interventions for one or more populations **Other criterial:** Written in English Original Research Studies published in or after 2013	**Population:** Non-European setting Studies involved other types of populations Studies focused on neurological diseases other than dementia Studies focused on dementia prevention before formal diagnosis **Intervention:** Study addressing other aspects of technology (e.g.; efficacy of a specific technology, performance tests, comparative test) Other study types do not meet specified criteria (e.g.; randomized controlled trials, case control, editorial, letters, conference paper). Studies not addressing attitudes toward technology or acceptability of technology or technological interventions **Other criterial:** Not written in English Not original research (e.g., editorial, opinion, consensus, systematic review, abstract) Studies published before 2013 Full text not available

### 2.2 Search strategy

PubMed was used as a primary dataset. Additional database searches were performed in the following databases: Embase, PsycINFO (EBSCOhost), Health Technology Assessment Database, and Web of Science (Clarivate). Duplicates were eliminated. These searches covered 10 years (2013–2023).

The research string employed was agreed by the team to ensure comprehensive coverage of relevant literature (Table 2). The keywords, aligned the PICO framework, include the following terms: Population (P) (“Alzheimer Disease” OR “Dementia”) AND Intervention/Outcome (I/O) (“Attitud*” OR “Perception” OR “Acceptanc*” OR “Digital” OR “Technolog*”) AND Geographical Area (S) (“Europ*” [MeSH]) AND Timeframe (T) (“2013/01/01”[PDAT]: “2023/12/31”[PDAT]). Additional relevant papers were manually searched for reference lists of collected studies and reviews. Gray literature such as conference papers, conference proceedings, dissertations, editorial letters, and other non-published documents that are not part of scientific journal publications, was not considered.

**Table 2:** Search terms used for papers’ identification.

Study population (P)	“Alzheimer Disease” OR “Dementia”
AND	
Intervention (I)	“Digital” OR “Technolog*”
AND	
Comparison (C)	Not Applicable
AND	
Outcome (O)	“Attitud*” OR “Perception” OR “Acceptanc*”
AND	
Geographical Area (S)	“Europ*” [MeSH]
AND	
Timeframe (T)	“2013/01/01”[PDAT]: “2023/12/31”[PDAT]

### 2.3 Data sources, studies sections, and data extraction

Five reviewers (MS, CF, MM, FE, IS) examined titles and abstracts of extracted articles, utilizing Rayyan Artificial Intelligence (32) to identify those adhering to the inclusion criteria. Duplicate entries were eliminated, and in cases where the abstracts lacked sufficient information to ascertain eligibility, full text review was performed. Conflicts and uncertainties during the article selection process were resolved through structured discussions. In cases of disagreement, collaborative review was conducted. If consensus could not be reached, the senior reviewer (RP) rendered the final decision, ensuring an impartial and standardized selection process.

### 2.4 Data analysis

The quality of the included papers was evaluated using the Mixed Methods Appraisal Tool (MMAT), revised version (33). This assessment tool evaluates various aspects of study quality based on the specific design of each study, taking into account the unique features of each type. The scores for the MMAT range from 0 to 100% depending on the study design criteria. Studies are not automatically excluded based on quality, but lower quality studies will be reviewed to determine their potential impact on the overall study results. In mixed methods studies, the overall quality of the combination cannot surpass the quality of its weakest component. Therefore, the overall quality score is determined by the lowest score among the study components.

The reviewers evaluated each paper independently to provide an objective assessment of the study quality.

## 3 Results

### 3.1 Study selection and characteristics

The initial search identified 1748 studies. Duplicates were removed using the Rayyan application, a database of 1,202 unique studies were compiled. Upon review of their titles and abstracts, 1,153 studies were deemed irrelevant and excluded. Subsequently, 49 publications underwent a full-text review, resulting in the selection of 13 studies meeting the inclusion criteria. Among these, 2 articles were retrieved from two reviews (34, 35) and included during the screening process. Gray literature was not considered, as well as conference papers, dissertations, letters, and editorials. The inclusion and exclusion criteria applied during this selection process are detailed in the Methods section, sub-section 2.1 Study design and summarized in Table 1. The 36 studies were excluded for the following reasons: 12 lacked assessment of attitudes toward technological intervention (including 1 case–control study and 1 case study), 14 were excluded due to their study design comparing two or more specific technologies in terms then acceptancy or attitude toward (including 4 randomized controlled trials, 1 case–control study), 4 were excluded for the type of study (2 study protocols, 2 systematic reviews), 5 were not focused on the population with Alzheimer’s and other dementias, and 1 study was excluded due to lack of full-text availability.

**Figure 1:**
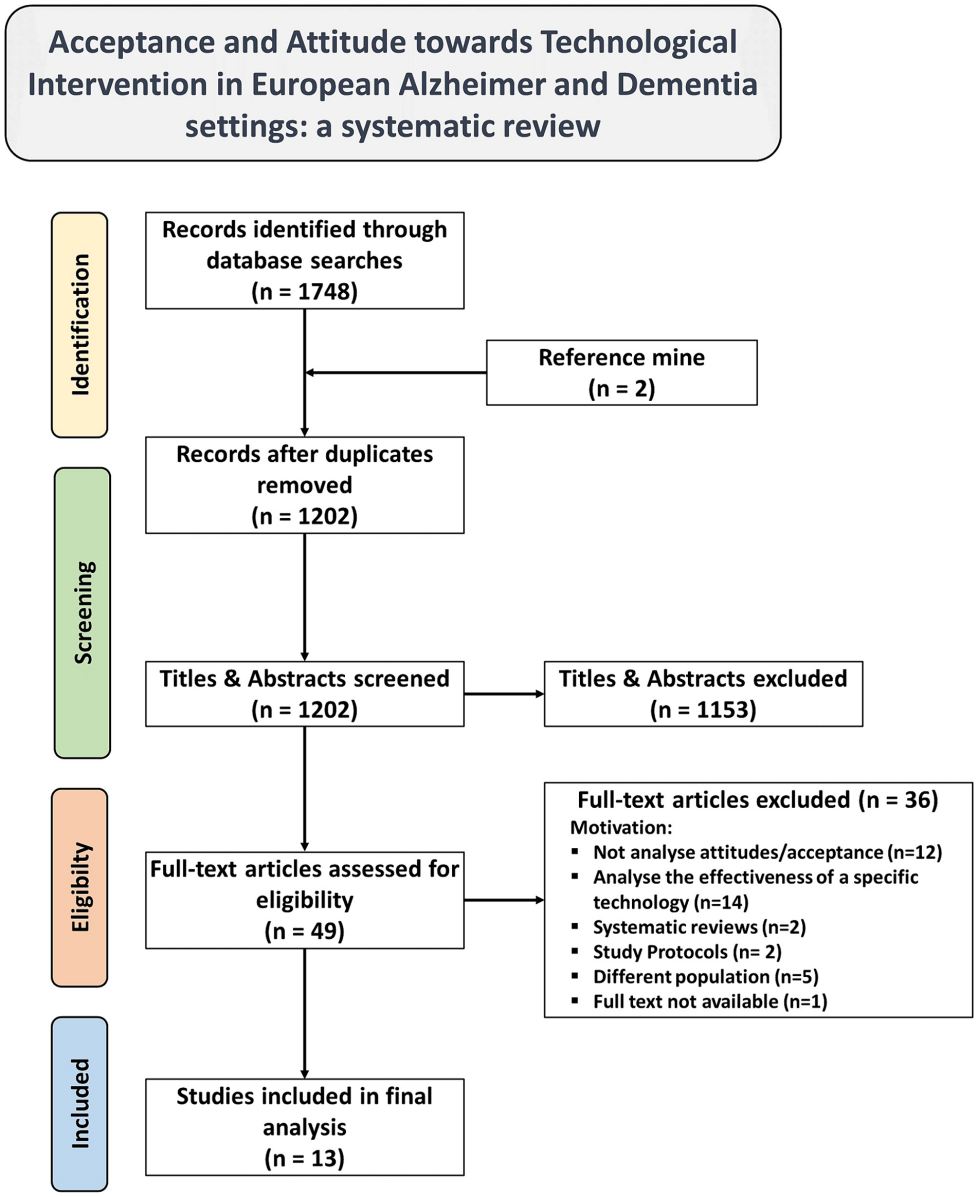
PRISMA flow diagram of literature search, abstract screen, full article assessment for exclusion and inclusion criteria with most common reasons for exclusion detailed.

A visual representation of this selection process and the reason for exclusion is provided in the PRISMA diagram (Figure 1). 13 studies, spanning 2014 to 2023 (36–48), were selected for this review. The publication years were: one study in 2014 (36), two in 2015 (37, 38), two in 2016 (39, 40); one in 2020 (41); three in 2021 (42–44); two in 2022 (45, 46); and two in 2023 (47, 48). Most of the studies were conducted primarily in United Kingdom (UK) (*n* = 6) (36–40, 42), across multiple European countries (*n* = 2) (41, 47), Germany (*n* = 2) (43, 45), in Italy (*n* = 1) (48) and the Netherlands (*n* = 1) (44). One study was conducted worldwide, encompassing Europe (*n* = 1) (46). The methodologies employed varied, including qualitative studies (*n* = 7) (37, 39, 42–44, 46, 48), mixed methods studies (*n* = 5) (36, 38, 40, 47), and quantitative studies (*n* = 1) (41, 45). These studies explored different types of technology, including locating technologies and remote monitoring (*n* = 4) (42, 43, 48, 49), digital technologies (*n* = 4) (36, 38, 40, 45), assistive technology (AT) (*n* = 3) (37, 39, 47), computer technologies (*n* = 1) (44), and attitudes toward technologies overall (*n* = 2) (41, 46). Sample sizes ranged from 6 to 2,172 participants. The focus of most papers was on PLWD and their caregivers (*n* = 4) (37, 38, 42, 46), while others concentrated solely on healthcare professionals (*n* = 2) (43, 45), key stakeholders (*n* = 1) (47), PLWD (*n* = 1) (40) and caregivers (*n* = 1) (36). Some studies (*n* = 2) targeted patients with cognitive impairments and their caregivers, with a subset focusing specifically on PLWD (41, 48). Two studies extended its focus to encompass PLWD, their caregivers, and healthcare professionals (39, 44). The characteristics of the articles included are summarized in Table 3.

**Table 3:** Characteristics of the included studies.

First author, year (cit.)	Nation	Study design & quality score	Population	Sample	Technology	Findings	Quality
McKechnie V et al., 2014 (36)	UK	Mixed-methods study	Caregivers	8 caregivers	Online support Forums	Interview participants reported a range of positive experiences and benefits from using the forum. Limited negative experiences were also reported	MMAT: 30%
Gibson G et al., 2015 (37)	UK	Qualitative study	PLWD and their caregivers	13 people dementia, 18 current family carers and 8 former carers (*n* = 39)	Assistive Technology	No-one in our study avoided the use of technology *per se*	MMAT: 100%
Tyack C et al., 2015 (38)	UK	Mixed-methods study	PLWD and their caregivers	Twelve pairs of volunteers with dementia and informal caregivers were recruited (*n* = 24)	Touchscreen-based art interventions	There was enthusiasm that using the app gave couples a new shared activity they could engage in, and all caregivers told us they enjoyed seeing their spouse becoming absorbed in viewing images.	MMAT: 30%
Newton L et al., 2016 (39)	UK	Qualitative study	Healthcare Professionals, PLWD and their caregivers	17 GPs, 13 people with dementia and 26 family carers (*n* = 56)	Assistive Technology	All participants had practical experience of witnessing AT being used in practice. For people with dementia and their families, knowledge was usually gained from personal experience rather than from health and social care professionals. For GPs, knowledge was largely gained through experiential, patient-led learning.	MMAT: 100%
Subramaniam P et al., 2016 (40)	UK	Mixed-methods study	PLWD	6 participants with mild to moderate dementia	Digital life storybooks	This format has been well received by all participants	MMAT: 30%
Guzman-Parra J Et al., 2020 (41)	Sweden, Spain	Quantitative Study	PLWD/mild cognitive impairment and caregivers	A total of 1,086 dyads were included (*N* = 2,172). Overall, 299 of people with dementia/mild cognitive impairment had a diagnosis of dementia.	Technophlia	The results of this study indicate that people with dementia/mild cognitive impairment have less technophilia than their caregivers	MMAT: 30%
Tuijt R et al., 2021 (42)	UK	Qualitative study	PLWD and their caregivers	30 people with dementia and 31 caregivers (*n* = 61)	Telemedicine	Attitudes toward technology varied, with some participants finding remote consultations adequate but many expressing a preference for in-person visits.	MMAT: 100%
Freiesleben SD et al., 2021 (43)	Germany	Qualitative study	Healthcare Professionals	Professionals working in business (*n* = 7), healthcare (*n* = 6) and research (*n* = 9) fields related to gerontology and gerontechnology (*n* = 22)	Locating technologies	he shared perception was that using locating technologies could result in increasing end-users’ quality of life on psychological, social, and physical levels	MMAT: 100%
van Gils AM et al., 2021 (44)	Netherlands	Qualitative study	Healthcare Professionals, PLWD and their caregivers	109 clinicians, 50 patients with subjective cognitive decline, mild cognitive impairment, or dementia and 46 care partners (*n* = 205)	Computer tools	Most clinicians reported a willingness to use diagnostic and prognostic computer tools; Patients and care partners were equally positive about the use of computer tools by clinicians, both for diagnosis and prognosis; most of them thought favorably regarding the possibility of using the tools themselves	MMAT: 100%
Hoel V et al., 2022 (45)	Germany	Mixed-methods study	Healthcare Professionals	409 facility managers and 8 directors of nursing (*n* = 417)	Digital devices	Attitude generally pessimistic about elderly PLWD being able to utilie technology independently	MMAT: 40%
Chirico I et al., 2022 (46)	UK, Italy, Australia, and Poland	Qualitative study	PLWD and their caregivers	15 people with dementia and 127 informal caregivers (*n* = 142)	Use of technology	Difficulties with remote healthcare arose, and teleconsultations, specifically specifically, seem not to work for peoplewith dementia who prefer physical contact and rely on non-verbal communication, especially when their cognitionis seriously compromised.	MMAT: 100%
Budak KB et al., 2023 (47)	Belgium, Bulgaria, Czech Republic, Finland, France, Germany, Greece, Luxembourg, Malta, the Netherlands, Norway, Portugal, Serbia, Slovenia, and Switzerland	Mixed method study	Stakeholders who were experts from European national and regional Alzheimer associations	11 participants from national Alzheimer associations, 13 participants from regional Alzheimer associations (*n* = 24)	Assisted Living Technology	Participants revealed a somewhat positive or negative attitude among persons living with dementia addressing loneliness	MMAT: 30%
Ruggiero F et al., 2023 (48)	Italy	Qualitative study	Patients with AD, amnesic MCI, FTD, LBD, mixed dementia, non-amnesic MCI, and MSA and caregivers	54 caregivers and 8 patients	Telemedicine	Telemedicine is well received by both	MMAT: 100%

### 3.2 Synthesized findings

Several studies reported a positive attitude toward various technological interventions in dementia care. For instance, a qualitative study involving key stakeholders from business, healthcare, and research fields highlighted a shared perception that using locating technologies could enhance the quality of life for PLWD (36). Additionally, a mixed-methods study focusing on caregivers of PLWD reported positive experiences from using online forums (36). Two studies introduced digital technologies, such as touchscreen-based art interventions and digital life storybooks, which received positive feedback from PLWD and their caregivers (38, 40). One study examined the perceptions of PLWD, current family caregivers, and former caregivers regarding AT, indicating a positive attitude (37). Similarly, a qualitative study conducted in the UK emphasized the crucial role of PLWD and their family caregivers in driving awareness and adoption of AT (39). Moreover, a qualitative study involving clinicians, patients with dementia, and care partners revealed a willingness to use diagnostic and prognostic computer tools (44).

During the COVID-19 pandemic, multiple studies were undertaken. A qualitative study emphasized the acceptance of telemedicine by both patients and caregivers (48). In a mixed-methods study, attitudes toward innovative technologies were examined among PLWD and caregivers, showing a decreased level of positive attitudes toward technology compared to their caregivers (41). Another study noted varied attitudes, with some participants regarding remote consultations as sufficient, while many expressed a preference for face-to-face (42). Additionally, a study involving stakeholders from European Alzheimer associations across 15 countries revealed both positive and negative attitudes among PLWD regarding Active assisted living (AAL) technology addressing loneliness (47). However, two additional studies underscored negative attitudes: a cross-sectional study among facility managers and directors of nursing revealed a generally pessimistic outlook regarding the ability of PLWD to utilize technology (45). Another study highlighted limited knowledge, technological unfamiliarity, and fear of the unknown among both patients and caregivers (49). Finally, a qualitative study highlighted the challenges in using telehealth, particularly teleconsultations, which appear ineffective for dementia patients preferring physical contact and relying on non-verbal communication, especially when their cognitive capacity is compromised (46).

## 4 Discussion

### 4.1 Summary of main findings

The intersection of technology and dementia care represents a dynamic field undergoing rapid evolution. Given the increasing burden of Alzheimer and dementia on both individuals and health systems, and recognizing the imperative to address the needs of those affected and their caregivers to tailor future interventions, this review sought to delve into the acceptance and attitudes toward technological interventions in European Alzheimer’s and dementia settings.

Thirteen studies, published from 2014 to 2023 were selected, showcasing a progressive engagement with this topic over the years. The distribution of publication years reveals a notable increase in studies from 2020 onwards, potentially reflecting a growing recognition of the importance of technological interventions in dementia care, particularly amongst the COVID-19 pandemic (50). The majority of studies were conducted in the UK, indicating a concentrated effort in this country to explore technological solutions for Alzheimer’s and other dementia care, given that during the pandemic, many dementia care services in the country faced challenges or substantial delays in implementing supportive technologies (51).

The selected studies employed a diverse range of methodologies, including qualitative, quantitative, and mixed-methods approaches. This methodological diversity reflects the complexity of technology implementation in dementia care, enriching the research by providing a more comprehensive understanding of the subject. The use of different approaches has allowed these studies to capture multiple dimensions of the challenges and opportunities in this area, providing deeper insights into both practical and theoretical aspects. This diversity highlights the value of integrating multiple perspectives to fully grasp the intricacies involved in dementia care technology. The focus of most papers was on PLWD and their caregivers, highlighting the centrality of their experiences and perspectives in shaping technological interventions in dementia care. Furthermore, several studies have focused on healthcare professionals and key stakeholders from the business, healthcare, and research sectors, highlighting the importance of involving these individuals. Indeed, the attitudes of healthcare professionals are positively impacted by computer literacy and familiarity with new technologies, indicating a necessity for training programs aimed at enhancing technological skills (52, 53).

Our results suggest a generally positive outlook toward technological interventions in dementia care across various studies. Positive attitudes were observed among PLWD, caregivers, healthcare professionals, highlighting the perceived benefits of locating and assistive technologies in enhancing the quality of life and promoting autonomy (37, 39, 43). Nevertheless, a study involving stakeholders from European Alzheimer associations across 15 countries revealed a spectrum of attitudes among PLWD toward AAL technology targeting loneliness, encompassing both positive and negative perspectives (47). However, it’s evident that technology can play a crucial role in promoting independent living, safety, and autonomy for people with dementia, thereby supporting their quality of life and that of their caregivers (54).

Moreover, digital activities were perceived favorably for enhancing communication among PLWD and caregivers (38, 40), addressing an important aspect of dementia care. In fact, caregivers and professionals encounter numerous challenges and barriers when communicating with PLWD, making effective communication essential for providing optimal care (55).

Another important finding was the acceptance of online forum to address social isolation among PLWD (36), even though they may face social challenges online, such as encountering stigmatizing language and negative comments, which may impact their well-being and deter them from going (56, 57).

Furthermore, attitudes toward computer technologies, such as diagnostic and prognostic computer tools, among healthcare professionals, patients with dementia, and care partners, were generally positive (44). Moreover, early detection of dementia not only alleviates distress for nursing staff and patients but also enhances health outcomes for patients (58, 59).

Notably, the COVID-19 pandemic brought to light both challenges and opportunities in the adoption of technology in dementia care. While some participants expressed reservations about remote consultations and preferred face-to-face interactions, others embraced telemedicine as a viable alternative during periods of restricted mobility and social distancing. The pandemic accelerated the adoption of telemedicine and digital solutions (60), leading to increased interest and acceptance among older adults with dementia (46, 48). However, it also revealed inequalities in attitudes toward technology, with some individuals exhibiting limited knowledge, technological unfamiliarity, and apprehension toward digital interventions (42). Notably, among facility managers and nursing directors, there was a prevailing pessimistic outlook regarding the ability of elderly individuals living with dementia to effectively utilize technology (45). This underscores the critical importance of addressing attitudes and perceptions to facilitate the integration of technology into dementia care, as the acceptance and adoption of new technological tools hinge significantly on how they are perceived by healthcare professionals (61).

Challenges arise from the symptoms of dementia, complicating the utilization of digital technologies (21). Additionally, there’s a noted lack of enthusiasm among general practitioners regarding telehealth, stemming from concerns about its potential impact on their workload (62, 63). Despite these challenges, the pandemic underscored the necessity of digitally mediated interactions for many (64). Such technologies have proven beneficial by enabling peer support, facilitating social interaction, and fostering a sense of identity (56, 65–67). Nevertheless, it is conceivable that the widespread reliance on digital technologies during the pandemic may have exacerbated pre-existing inequalities, leading to a deepened digital divide (41, 68).

In conclusion, the examination of attitudes toward innovative technologies among PLWD and their caregivers revealed notable differences, with caregivers exhibiting a higher level of positive attitude compared with individuals with dementia themselves, mainly due to the lack of technology enthusiasm (41). This could be not necessarily due to technology, but could as well stem from apathy, commonly associated with dementia and mild cognitive impairment, and related behavioral symptoms (69, 70). Apathy could potentially hinder enthusiasm toward new technologies and serve as a barrier for interventions requiring learning and adaptation to their use.

The research shows positive attitudes toward technology in dementia, but also emphasizes the need for interventions, education, and support for all stakeholders, including patients and professionals, underscoring the importance of considering the perspectives and preferences of all stakeholders when designing and implementing technological interventions in dementia care.

### 4.2 Strengths and limitations

This systematic review possesses several strengths that enhance the robustness of its findings. Firstly, adherence to the Preferred Reporting Items for Systematic Reviews and Meta-Analyses (PRISMA) guidelines ensures transparency and rigor in the review process, minimizing bias and enhancing reproducibility. Additionally, the comprehensive search strategy encompassing multiple databases over a 10-year period maximizes the retrieval of relevant literature, providing a comprehensive overview of the topic. Moreover, the involvement of multiple reviewers and the utilization of Rayyan Artificial Intelligence for screening enhance the reliability of study selection and minimize errors. Finally, the systematic assessment of the methodological quality of included studies further strengthens the validity of the review’s findings by identifying potential biases and limitations in individual studies.

However, despite these strengths, there are limitations to acknowledge. Firstly, the restriction to studies published in English and conducted in Europe may introduce language and geographical biases, potentially overlooking valuable insights from non-English literature or studies conducted in other regions. Even though one study was conducted also on non-European countries, it also encompassed European countries, we decided to include it. This decision was made since, although some of the countries analyzed were non-European, the paper did maintain the granularity of the data allowing us to better understand the impact of technological interventions and attitudes within the European context. Another potential limitation is the inclusion of studies conducted in the UK. Although the UK is no longer part of the European Union, its geographical proximity and historical integration within European research frameworks, as well as the similarity of the UK Health System organization with the majority of the other European Health Systems, make it relevant for understanding broader trends in the European context. Additionally, the exclusion of studies reporting a comparation between two or more assistive technologies, might have limited the breadth of evidence considered, particularly regarding the efficacy of technological interventions. Nonetheless the efficacy of assistive technologies is beyond the scope of this review and none of those studies addressed the perspective of either attitudes or acceptability. Furthermore, the reliance on published literature may lead to publication bias, where studies with positive results are more likely to be published, potentially skewing the overall findings.

Moreover, significant methodological diversity among the included studies presents another limitation. The heterogeneity in study designs, measurement tools, and outcome assessments may have impacted the review results and conclusions. Such heterogeneity can pose a challenge in summarizing the findings and may affect the generalizability of the results. Despite efforts to systematically assess the quality of included studies, this variability could introduce inconsistencies that influence the overall interpretation of the data. Another key limitation of this review is the absence of an in-depth analysis of specific types of technologies used in dementia care, despite the inclusion of various devices such as location tools, digital technologies, and assistive technologies. Consequently, the review may lack the desired level of detail regarding the factors influencing acceptance of specific technologies. Future research could address this by providing a more detailed examination of how different technologies are perceived and utilized by patients, caregivers, and healthcare professionals, tailored to specific needs and contexts. Finally, the timeframe of the search, covering only the years 2013–2023, may omit relevant studies published before or after this period, potentially impacting the comprehensiveness of the review. Despite these limitations, the systematic review provides valuable insights into the acceptability and attitudes toward technological interventions in European patients with Alzheimer’s or other dementias, offering guidance for future research and clinical practice.

## 5 Conclusion

The attitude and acceptance of technology hold significant importance in the context of dementia care. This demographic shift toward embracing technology reflects a fundamental change in how individuals interact with their environment and manage their health.

Understanding and addressing both aspects—attitude and acceptance—toward technology in dementia settings is crucial for several reasons. Firstly, they influence the adoption and utilization of technology-based interventions aimed at improving dementia care. Positive attitudes can facilitate the integration of technological solutions into daily routines, enhancing their effectiveness in managing symptoms and improving quality of life. Conversely, negative attitudes can hinder adoption, leading to underutilization of potentially beneficial tools and services.

Acceptance of technology, however, extends beyond mere willingness; it involves the practical integration and use of these technologies. Factors such as familiarity with the technology, perceived usefulness, and adaptability to the specific needs of people living with dementia (PLWD) are critical for successful acceptance. Embracing technology can empower individuals to maintain their independence, engage in meaningful activities, and stay connected with their communities. Conversely, reluctance or resistance may limit access to essential resources and support networks, exacerbating feelings of isolation and dependency.

By recognizing and addressing both attitudes and practical acceptance of technology in dementia care, healthcare providers, policymakers, and technology developers can tailor interventions to meet the diverse needs of this population. Strategies such as user-centered design, education, and training can alleviate concerns, build confidence, and promote effective use of technology.

Such initiatives not only empower PLWD but also contribute to fostering a more inclusive and technologically literate society.

## Data Availability

The original contributions presented in the study are included in the article/supplementary material, further inquiries can be directed to the corresponding author.

## References

[1] Li K Li A Mei Y Zhao J Zhou Q Li Y . Trace elements and Alzheimer dementia in population-based studies: a bibliometric and meta-analysis. Environ Pollut. (2023) 318:120782. doi: 10.1016/j.envpol.2022.120782, PMID: 36464120

[2] Nichols E Steinmetz JD Vollset SE Fukutaki K Chalek J Abd-Allah F . Estimation of the global prevalence of dementia in 2019 and forecasted prevalence in 2050: an analysis for the global burden of disease study 2019. Lancet Public Health. (2022) 7:e105–25. doi: 10.1016/S2468-2667(21)00249-8, PMID: 34998485 PMC8810394

[3] Georges J Miller O Bintener C (2020) Estimating the prevalence of dementia in Europe. Alzheimer Europe.

[4] Wolters FJ Chibnik LB Waziry R Anderson R Berr C Beiser A . Twenty-seven-year time trends in dementia incidence in Europe and the United States. Neurology. (2020) 95:e519–31. doi: 10.1212/WNL.0000000000010022, PMID: 32611641 PMC7455342

[5] Matthews FE Stephan BCM Robinson L Jagger C Barnes LE Arthur A . A two decade dementia incidence comparison from the cognitive function and ageing studies I and II. Nat Commun. (2016) 7:11398. doi: 10.1038/ncomms11398, PMID: 27092707 PMC4838896

[6] World Health Organization. Dementia. (2023). Available at: https://www.who.int/news-room/fact-sheets/detail/dementia (Accessed June 6, 2024).

[7] Thordardottir B Malmgren Fänge A Lethin C Rodriguez Gatta D Chiatti C. Acceptance and use of innovative assistive technologies among people with cognitive impairment and their caregivers: a systematic review. Biomed Res Int. (2019) 2019:1–18. doi: 10.1155/2019/9196729, PMID: 30956989 PMC6431399

[8] Dyer SM Harrison SL Laver K Whitehead C Crotty M. An overview of systematic reviews of pharmacological and non-pharmacological interventions for the treatment of behavioral and psychological symptoms of dementia. Int Psychogeriatr. (2018) 30:295–309. doi: 10.1017/S1041610217002344 29143695

[9] Leng M Zhao Y Xiao H Li C Wang Z. Internet-based supportive interventions for family caregivers of people with dementia: systematic review and Meta-analysis. J Med Internet Res. (2020) 22:e19468. doi: 10.2196/19468, PMID: 32902388 PMC7511858

[10] Ghafurian M Hoey J Dautenhahn K. Social robots for the Care of Persons with dementia. ACM Trans Hum Robot Interact. (2021) 10:1–31. doi: 10.1145/3469653

[11] Alves GS Casali ME Veras AB Carrilho CG Bruno Costa E Rodrigues VM . A systematic review of home-setting psychoeducation interventions for behavioral changes in dementia: some lessons for the COVID-19 pandemic and post-pandemic assistance. Front Psych. (2020) 11:577871. doi: 10.3389/fpsyt.2020.577871, PMID: 33132937 PMC7550734

[12] Peek STM Wouters EJM van Hoof J Luijkx KG Boeije HR Vrijhoef HJM. Factors influencing acceptance of technology for aging in place: a systematic review. Int J Med Inform. (2014) 83:235–48. doi: 10.1016/j.ijmedinf.2014.01.004, PMID: 24529817

[13] Sanders D Scott P. Literature review: technological interventions and their impact on quality of life for people living with dementia. BMJ Health Care Inform. (2020) 27:e100064. doi: 10.1136/bmjhci-2019-100064, PMID: 31948938 PMC7062354

[14] Huisman C Huisman E Kort H. Technological applications contributing to relieve care burden or to sleep of caregivers and people with dementia: a scoping review from the perspective of social isolation. Front Public Health. (2022) 10:797176. doi: 10.3389/fpubh.2022.797176, PMID: 35425752 PMC9002108

[15] Heinz M Martin P Margrett JA Yearns M Franke W Yang HI . Perceptions of technology among older adults. J Gerontol Nurs. (2013) 39:42–51. doi: 10.3928/00989134-20121204-04 23244061

[16] Tung F-C Chang S-C. Exploring adolescents’ intentions regarding the online learning courses in Taiwan. Cyberpsychol Behav. (2007) 10:729–30. doi: 10.1089/cpb.2007.9960, PMID: 17927546

[17] Zhang M. Older people’s attitudes towards emerging technologies: a systematic literature review. Public Underst Sci. (2023) 32:948–68. doi: 10.1177/09636625231171677, PMID: 37204075 PMC10631270

[18] Alshahrani A Stewart D MacLure K. A systematic review of the adoption and acceptance of eHealth in Saudi Arabia: views of multiple stakeholders. Int J Med Inform. (2019) 128:7–17. doi: 10.1016/j.ijmedinf.2019.05.007, PMID: 31160014

[19] Wei G Diehl-Schmid J Matias-Guiu JA Pijnenburg Y Landin-Romero R Bogaardt H . The effects of the COVID-19 pandemic on neuropsychiatric symptoms in dementia and carer mental health: an international multicentre study. Sci Rep. (2022) 12:2418. doi: 10.1038/s41598-022-05687-w, PMID: 35165292 PMC8844310

[20] Talbot CV Briggs P. ‘Getting back to normality seems as big of a step as going into lockdown’: the impact of the COVID-19 pandemic on people with early to middle stage dementia. Age Ageing. (2021) 50:657–63. doi: 10.1093/ageing/afab012, PMID: 33481988 PMC7929391

[21] Giebel C Cannon J Hanna K Butchard S Eley R Gaughan A . Impact of COVID-19 related social support service closures on people with dementia and unpaid carers: a qualitative study. Aging Ment Health. (2021) 25:1281–8. doi: 10.1080/13607863.2020.1822292, PMID: 32954794

[22] Tondo G Sarasso B Serra P Tesser F Comi C. The impact of the COVID-19 pandemic on the cognition of people with dementia. Int J Environ Res Public Health. (2021) 18:4285. doi: 10.3390/ijerph18084285 33919491 PMC8073614

[23] Willis E Semple AC de Waal H. Quantifying the benefits of peer support for people with dementia: a social return on investment (SROI) study. Dementia. (2018) 17:266–78. doi: 10.1177/1471301216640184, PMID: 27013520

[24] Giebel CM Challis DJ Montaldi D. A revised interview for deterioration in daily living activities in dementia reveals the relationship between social activities and well-being. Dementia. (2016) 15:1068–81. doi: 10.1177/1471301214553614, PMID: 25280491

[25] Canevelli M Valletta M Toccaceli Blasi M Remoli G Sarti G Nuti F . Facing dementia during the <scp>COVID</scp> −19 outbreak. J Am Geriatr Soc. (2020) 68:1673–6. doi: 10.1111/jgs.16644, PMID: 32516441 PMC7300919

[26] Spreadbury JH Kipps C. Measuring younger onset dementia: what the qualitative literature reveals about the ‘lived experience’ for patients and caregivers. Dementia. (2019) 18:579–98. doi: 10.1177/1471301216684401, PMID: 28114802

[27] Giebel C Pulford D Cooper C Lord K Shenton J Cannon J . COVID-19-related social support service closures and mental well-being in older adults and those affected by dementia: a UK longitudinal survey. BMJ Open. (2021) 11:e045889. doi: 10.1136/bmjopen-2020-045889, PMID: 33455941 PMC7813330

[28] Neal D van den Berg F Planting C Ettema T Dijkstra K Finnema E . Can use of digital technologies by people with dementia improve self-management and social participation? A systematic review of effect studies. J Clin Med. (2021) 10:604. doi: 10.3390/jcm10040604, PMID: 33562749 PMC7915697

[29] Peek STM Wouters EJ Luijkx KG Vrijhoef HJ. What it takes to successfully implement technology for aging in place: focus groups with stakeholders. J Med Internet Res. (2016) 18:e98. doi: 10.2196/jmir.5253, PMID: 27143097 PMC4904824

[30] Cook EJ Randhawa G Guppy A Sharp C Barton G Bateman A . Exploring factors that impact the decision to use assistive telecare: perspectives of family care-givers of older people in the United Kingdom. Ageing Soc. (2018) 38:1912–32. doi: 10.1017/S0144686X1700037X

[31] Liberati A Altman DG Tetzlaff J Mulrow C Gøtzsche PC Ioannidis JPA . The PRISMA statement for reporting systematic reviews and meta-analyses of studies that evaluate health care interventions: explanation and elaboration. J Clin Epidemiol. (2009) 62:e1–e34. doi: 10.1016/j.jclinepi.2009.06.006, PMID: 19631507

[32] Rayyan. Intelligent systematic review. (2022). Available at: https://www.rayyan.ai/

[33] Hong QN Fàbregues S Bartlett G Boardman F Cargo M Dagenais P . The mixed methods appraisal tool (MMAT) version 2018 for information professionals and researchers. Educ Inf. (2018) 34:285–91. doi: 10.3233/EFI-180221

[34] Neal I du Toit SHJ Lovarini M. The use of technology to promote meaningful engagement for adults with dementia in residential aged care: a scoping review. Int Psychogeriatr. (2020) 32:913–35. doi: 10.1017/S1041610219001388, PMID: 31547900

[35] García-Vivar C Konradsen H Kolbrun Svavarsdóttir E Brødsgaard A Dieperink KB Luttik ML . Healthcare interventions for older people with dementia and family caregivers in Europe: a scoping review. Int J Nurs Pract. (2024) 30:e13172. doi: 10.1111/ijn.13172, PMID: 37287366

[36] McKechnie V Barker C Stott J. The effectiveness of an internet support forum for carers of people with dementia: a pre-post cohort study. J Med Internet Res. (2014) 16:e68. doi: 10.2196/jmir.3166, PMID: 24583789 PMC3961748

[37] Gibson G Dickinson C Brittain K Robinson L. The everyday use of assistive technology by people with dementia and their family carers: a qualitative study. BMC Geriatr. (2015) 15:89. doi: 10.1186/s12877-015-0091-3, PMID: 26205957 PMC4514453

[38] Tyack C Camic PM Heron MJ Hulbert S. Viewing art on a tablet computer: a well-being intervention for people with dementia and their caregivers. J Appl Gerontol. (2017) 36:864–94. doi: 10.1177/0733464815617287, PMID: 26675353

[39] Newton L Dickinson C Gibson G Brittain K Robinson L. Exploring the views of GPs, people with dementia and their carers on assistive technology: a qualitative study: table 1. BMJ Open. (2016) 6:e011132. doi: 10.1136/bmjopen-2016-011132, PMID: 27178978 PMC4874138

[40] Woods B Subramaniam P. Digital life storybooks for people with dementia living in care homes: an evaluation. Clin Interv Aging. (2016) 11:1263–76. doi: 10.2147/CIA.S111097, PMID: 27698556 PMC5034922

[41] Guzman-Parra J Barnestein-Fonseca P Guerrero-Pertiñez G Anderberg P Jimenez-Fernandez L Valero-Moreno E . Attitudes and use of information and communication technologies in older adults with mild cognitive impairment or early stages of dementia and their caregivers: cross-sectional study. J Med Internet Res. (2020) 22:e17253. doi: 10.2196/17253 32442136 PMC7296403

[42] Tuijt R Rait G Frost R Wilcock J Manthorpe J Walters K. Remote primary care consultations for people living with dementia during the COVID-19 pandemic: experiences of people living with dementia and their carers. Br J Gen Pract. (2021) 71:e574–82. doi: 10.3399/BJGP.2020.1094, PMID: 33630749 PMC8136581

[43] Freiesleben SD Megges H Herrmann C Wessel L Peters O. Overcoming barriers to the adoption of locating technologies in dementia care: a multi-stakeholder focus group study. BMC Geriatr. (2021) 21:378. doi: 10.1186/s12877-021-02323-6, PMID: 34154542 PMC8218472

[44] van Gils AM Visser LN Hendriksen HM Georges J Muller M Bouwman FH . Assessing the views of professionals, patients, and care partners concerning the use of computer tools in memory clinics: international survey study. JMIR Form Res. (2021) 5:e31053. doi: 10.2196/31053, PMID: 34870612 PMC8686488

[45] Hoel V Seibert K Domhoff D Preuß B Heinze F Rothgang H . Social health among German nursing home residents with dementia during the COVID-19 pandemic, and the role of technology to promote social participation. Int J Environ Res Public Health. (2022) 19:1956. doi: 10.3390/ijerph19041956, PMID: 35206143 PMC8872488

[46] Chirico I Giebel C Lion K Mackowiak M Chattat R Cations M . Use of technology by people with dementia and informal carers during COVID-19: a cross-country comparison. Int J Geriatr Psychiatry. (2022) 37:1–10. doi: 10.1002/gps.5801, PMID: 36005276

[47] Budak KB Laporte Uribe F Meiland F Felding SA Teupen S Bergmann JM . Implementing active assisted living technology in the long-term care of people living with dementia to address loneliness: European survey. JMIR Aging. (2023) 6:e45231. doi: 10.2196/45231, PMID: 37314840 PMC10334712

[48] Ruggiero F Zirone E Molisso MT Carandini T Fumagalli G Pietroboni A . Telemedicine for cognitive impairment: a telephone survey of patients’ experiences with neurological video consultation. Neurol Sci. (2023) 44:3885–94. doi: 10.1007/s10072-023-06903-9, PMID: 37365397 PMC10570200

[49] Arighi A Fumagalli GG Carandini T Pietroboni AM de Riz MA Galimberti D . Facing the digital divide into a dementia clinic during COVID-19 pandemic: caregiver age matters. Neurol Sci. (2021) 42:1247–51. doi: 10.1007/s10072-020-05009-w, PMID: 33459891 PMC7811944

[50] Barbosa A Ferreira AR Smits C Hegerath FM Vollmar HC Fernandes L . Use and uptake of technology by people with dementia and their supporters during the COVID-19 pandemic. Aging Ment Health. (2024) 28:83–94. doi: 10.1080/13607863.2022.2163375 36650751

[51] Giebel C Hanna K Callaghan S Cannon J Butchard S Shenton J . Navigating the new normal: accessing community and institutionalised care for dementia during COVID-19. Aging Ment Health. (2022) 26:905–10. doi: 10.1080/13607863.2021.1914545, PMID: 33908284

[52] Ifinedo P. The moderating effects of demographic and individual characteristics on nurses’ acceptance of information systems: a Canadian study. Int J Med Inform. (2016) 87:27–35. doi: 10.1016/j.ijmedinf.2015.12.012, PMID: 26806709

[53] Ifinedo P. Empirical study of Nova Scotia nurses’ adoption of healthcare information systems: implications for management and policy-making. Int J Health Policy Manag. (2017) 7:317–27. doi: 10.15171/ijhpm.2017.96, PMID: 29626399 PMC5949222

[54] Van der Roest HG Wenborn J Pastink C Dröes RM Orrell M. Assistive technology for memory support in dementia. Cochrane Database Syst Rev. (2017) 2017:CD009627. doi: 10.1002/14651858.CD009627.pub2, PMID: 28602027 PMC6481376

[55] Nguyen H Eccleston CE Doherty KV Jang S McInerney F. Communication in dementia care: experiences and needs of carers. Dementia. (2022) 21:1381–98. doi: 10.1177/14713012221080003 35333128

[56] Talbot CV O’Dwyer ST Clare L Heaton J. The use of twitter by people with young-onset dementia: a qualitative analysis of narratives and identity formation in the age of social media. Dementia. (2021) 20:2542–57. doi: 10.1177/14713012211002410, PMID: 33765848 PMC8564236

[57] Oscar N Fox PA Croucher R Wernick R Keune J Hooker K. Machine learning, sentiment analysis, and tweets: an examination of Alzheimer’s disease stigma on twitter. J Gerontol Ser B. (2017) 72:742–51. doi: 10.1093/geronb/gbx014, PMID: 28329835

[58] Hessler JB Schäufele M Hendlmeier I Junge MN Leonhardt S Weber J . Behavioural and psychological symptoms in general hospital patients with dementia, distress for nursing staff and complications in care: results of the general hospital study. Epidemiol Psychiatr Sci. (2018) 27:278–87. doi: 10.1017/S2045796016001098, PMID: 28065176 PMC6998873

[59] Sampson EL White N Lord K Leurent B Vickerstaff V Scott S . Pain, agitation, and behavioural problems in people with dementia admitted to general hospital wards: a longitudinal cohort study. Pain. (2015) 156:675–83. doi: 10.1097/j.pain.0000000000000095, PMID: 25790457 PMC4381983

[60] Golinelli D Boetto E Carullo G Nuzzolese AG Landini MP Fantini MP. Adoption of digital technologies in health care during the COVID-19 pandemic: systematic review of early scientific literature. J Med Internet Res. (2020) 22:e22280. doi: 10.2196/22280, PMID: 33079693 PMC7652596

[61] Safi S Thiessen T Schmailzl KJ. Acceptance and resistance of new digital technologies in medicine: qualitative study. JMIR Res Protoc. (2018) 7:e11072. doi: 10.2196/11072, PMID: 30514693 PMC6299231

[62] Segar J Rogers A Salisbury C Thomas C. Roles and identities in transition: boundaries of work and inter-professional relationships at the interface between telehealth and primary care. Health Soc Care Commun. (2013) 21:606–13. doi: 10.1111/hsc.12047, PMID: 23656381

[63] MacNeill V Sanders C Fitzpatrick R Hendy J Barlow J Knapp M . Experiences of front-line health professionals in the delivery of telehealth: a qualitative study. Br J Gen Pract. (2014) 64:e401–7. doi: 10.3399/bjgp14X680485, PMID: 24982492 PMC4073725

[64] Beaunoyer E Dupéré S Guitton MJ. COVID-19 and digital inequalities: reciprocal impacts and mitigation strategies. Comput Human Behav. (2020) 111:106424. doi: 10.1016/j.chb.2020.106424, PMID: 32398890 PMC7213963

[65] Talbot CV O’Dwyer ST Clare L Heaton J Anderson J. How people with dementia use twitter: a qualitative analysis. Comput Human Behav. (2020) 102:112–9. doi: 10.1016/j.chb.2019.08.005

[66] Kannaley K Mehta S Yelton B Friedman DB. Thematic analysis of blog narratives written by people with Alzheimer’s disease and other dementias and care partners. Dementia. (2019) 18:3071–90. doi: 10.1177/1471301218768162, PMID: 29642716 PMC6027602

[67] Craig D Strivens E. Facing the times: a young onset dementia support group: Facebook TM style. Australas J Ageing. (2016) 35:48–53. doi: 10.1111/ajag.12264, PMID: 27010874

[68] Lai J Widmar NO. Revisiting the digital divide in the <scp>COVID</scp> −19 era. Appl Econ Perspect Policy. (2021) 43:458–64. doi: 10.1002/aepp.13104 33230409 PMC7675734

[69] van der Linde RM Matthews FE Dening T Brayne C. Patterns and persistence of behavioural and psychological symptoms in those with cognitive impairment: the importance of apathy. Int J Geriatr Psychiatry. (2017) 32:306–15. doi: 10.1002/gps.4464, PMID: 27017917

[70] Sherman C Liu CS Herrmann N Lanctôt KL. Prevalence, neurobiology, and treatments for apathy in prodromal dementia. Int Psychogeriatr. (2018) 30:177–84. doi: 10.1017/S1041610217000527, PMID: 28416030

